# Voltammetric Determination of Isoniazid in the Presence of Acetaminophen Utilizing MoS_2_-Nanosheet-Modified Screen-Printed Electrode

**DOI:** 10.3390/mi13030369

**Published:** 2022-02-26

**Authors:** Somayeh Tajik, Zahra Dourandish, Fariba Garkani Nejad, Abbas Aghaei Afshar, Hadi Beitollahi

**Affiliations:** 1 Research Center of Tropical and Infectious Diseases, Kerman University of Medical Sciences, Kerman 7616913555, Iran; tajik_s1365@yahoo.com (S.T.); abassaghaeiafshar@gmail.com (A.A.A.); 2 Environment Department, Institute of Science and High Technology and Environmental Sciences, Graduate University of Advanced Technology, Kerman 7631885356, Iran; z.dourandish2017@gmail.com (Z.D.); f.garkani95@gmail.com (F.G.N.)

**Keywords:** isoniazid, acetaminophen, MoS_2_ nanosheets, voltammetric sensors, modified electrode

## Abstract

We used MoS_2_ nanosheets (MoS_2_ NSs) for surface modification of screen-printed electrode (MoS_2_NSs-SPE) aimed at detecting isoniazid (INZ) in the presence of acetaminophen (AC). According to analysis, an impressive catalytic performance was found for INZ and AC electro-oxidation, resulting in an appreciable peak resolution (~320 mV) for both analytes. Chronoamperometry, differential pulse voltammetry (DPV), linear sweep voltammogram (LSV), and cyclic voltammetry (CV) were employed to characterize the electrochemical behaviors of the modified electrode for the INZ detection. Under the optimal circumstances, there was a linear relationship between the peak current of oxidation and the various levels of INZ (0.035–390.0 µM), with a narrow limit of detection (10.0 nM). The applicability of the as-developed sensor was confirmed by determining the INZ and AC in tablets and urine specimens, with acceptable recoveries.

## 1. Introduction

Drug analysis as one of the main branches of analytical chemistry is essential to control the quality of drugs. Drug analysis has used a variety of analytical techniques such as high-performance liquid chromatography (HPLC) [1,2], mass spectrometry [3], liquid chromatography–mass spectrometry/mass-spectrometry (LC–MS/MS) [4], and chemiluminescence [5,6,7]. Despite the many advantages of all of these methods, there are some drawbacks such as sophisticated analysis, high cost, low sensitivity, and long response times.

Analytical approaches based on electrochemical sensing systems possess multiple merits such as cost-effectiveness, portability, simple devices, narrow limit of detection (LOD), high-speed analysis, extended linear dynamic range, and selectivity in exposure to interferants [8,9,10,11,12]. Voltammetric determinations, such as DPV, exhibit rapid reaction, excellent sensitivity, and impressive selectivity [13,14,15].

Screen-printing electrodes (SPEs) have been extensively employed for the mass-production of disposable electrochemical sensing systems [16]. The SPEs are affordable with the capability for mass production while maintaining sufficient reproducibility, with advantages of versatility and miniaturization [17,18,19,20,21]. The electrocatalytic activity of the bare electrode displays very substandard behavior [22]. Therefore, the electrode surface modification increases the sensitivity, reproducibility, and stability [23,24,25,26,27]. Detection of trace level of analytes can be increased by combining nanomaterials, significantly reinforcing the surface properties and electroconductivity of the electrodes, from such a function [28,29,30,31]. Nanomaterial-supported electrochemical sensors have recently attracted the attention of researchers [32,33,34].

Two-dimensional (2D) layered nanomaterials such as transition metal dichalcogenides (TMDs) and graphene are of the ideal electrode surface modifiers because of specific physicochemical features [35]. Molybdenum disulfide (MoS_2_) is one of the key TMDs, having an impressive layered structure, which contains monolayer of Mo atoms located within two layers of S atoms in a trigonal prismatic arrangement. These sandwich layers in the crystalline lattice are linked together by weak Van der Waals forces, such that the distance of inter-sheet molybdenum atoms are 0.65 nm [36,37,38]. A silicon-like semiconductor of bulk MoS_2_ has an indirect 1.23-eV bandgap, and the two-dimensional sheets of MoS_2_ (2D-MoS_2_) form a direct 1.89-eV bandgap (visible red) due to the removal of interplays between layers and confining electrons in a single plane. Furthermore, MoS_2_ nanosheets possess numerous unparalleled features, including impressive biocompatibility, huge surface area, durable structural stability, and appreciable junction area with electrode/reactants. Such structural merits and resultant electrical and optical features suggest the applicability of 2D-MoS_2_ NSs and other 2D-TMDs to develop various optoelectronic and electronic equipment such as photodetectors, field-effect transistors (FETs), varied sensors, and photovoltaic devices [39,40,41,42].

N-acetyl-p-aminophenol, known as acetaminophen, paracetamol, Tylenol, or AC, is a drug globally used for relieving moderate pain and declining fever. AC is a noncarcinogenic and effective aspirin substitute for people with hypersensitivity to acetylsalicylic acid [43,44]. The analgesic pathway of AC is to impede production of prostaglandin in the central nervous system (CNS). It can reduce fever through sedation of the hypothalamic heat-regulating center. This drug is mainly metabolized in the liver where toxic metabolites are produced. The overdose of AC (about 4 g/day) can result in side effects such as hepatoxicity, nephrotoxicity, gastrointestinal problems, and tissue failures [45,46].

The contagious infection of tuberculosis (TB) caused by *Mycobacterium tuberculosis* (MB-TB) bacteria can influence various body organs, in particular the lungs. Pyridine-4-carboxylic acid hydrazide, called an isonicotinic acid hydrazide, isoniazid, or INZ, is an essential organic compound and a beneficial antibiotic capable of exhibiting strong bactericidal performance in the early steps of anti-TB therapy [47,48]. Many new cases of tuberculosis are treated each year with isoniazid as the most effective and the safest treatment. TB-related medications should be prescribed for a longer period of time. It should be noted that INZ may induce hepatotoxicity in people suffering from inflammation and may even cause death after long-term exposure to INZ [49,50]. Since antibacterials (INZ) and AC are routine, commercially available medications, a growing concern has been aroused over liver damage because of INZ co-administrated with AC [51]. Accordingly, it is substantial to develop a sensitive and selective electrochemical sensor in pharmaceutical and clinical preparations to detect INZ co-administrated with AC.

Herein, we used MoS_2_ NSs for surface modification of a screen-printed electrode aimed at simultaneously and voltammetrically detecting INZ and its common interferant AC. The MoS_2_ NSs were synthesized by a single-pot hydrothermal protocol. Thus, drop-casting of dispersed MoS_2_ NSs on SPE surfaces leads to the preparation of sensors (MoS_2_NSs-SPE). These sensors had a greater electrocatalytic response to detecting INZ in buffer solution (pH = 7.0) when compared with bare (unmodified) SPEs. The applicability of the as-developed sensor was confirmed by determining the INZ and AC in real tablets and urine specimens, with acceptable recoveries.

## 2. Experimental

### 2.1. Materials and Instrumentations

A potentiostat/galvanostat AUTOLAB PGSTAT 302N (Metrohm, Herisau, Switzerland) was utilized to carry out all experiments during the electrochemical processes, under monitoring of the General Purpose Electrochemical System (GPES) software Version 4.9. The DropSens SPE (DRP-110, Oviedo, Spain) was used for all electrochemical tests. The three-electrode composition contained a 4 mm graphite as the working electrode, graphite as the auxiliary electrode, and a silver as pseudo-reference electrode. A Metrohm 713 pH meter (Metrohm, Herisau, Switzerland) equipped with a glass electrode was utilized to measure the pH values of all solutions. Deionized water from Direct-Q^®®^ 8 UV water purification system (Millipore, Darmstadt, Germany) was applied to freshly prepare all solutions. X-ray diffraction (XRD) spectra were obtained from Panalytical X’Pert Pro X-ray diffractometer (Etten Leur, The Netherlands) with Cu/Ká radiation at ë value of 1.5418 nm. Fourier-transform infrared (FTIR) patterns were obtained from a Tensor II spectrometer (Bruker, Mannheim, Germany). Energy dispersive X-ray (EDX) patterns and scanning electron microscopy (SEM) images were obtained by the MIRA3 scanning electron microscope (Tescan, Brno, Czech Republic).

All materials in this study were of analytical grade with no extra purification, sourced from Sigma-Aldrich. Phosphoric acid was utilized to prepare phosphate buffer solutions (PBSs) with various pH values adjusted by NaOH.

### 2.2. Fabrication of MoS_2_ NSs

Based on a protocol, (NH_4_)_6_Mo_7_O_24_·4H_2_O (3 mmol) and thiourea (2.3 g) dispersed in deionized water (30 mL) were transferred to a Teflon autoclave (40-mL) at 200 °C for 24 h. The resultant product was adequately rinsed with ethanol and deionized water, followed by vacuum drying at 50 °C for six hours.

### 2.3. Preparation of MoS_2_ NSs-SPE

For the preparation of MoS_2_ NSs-SPE, 1 mg of synthesized MoS_2_ NSs was poured into 1 mL of deionized water under ultrasonication, followed by drop casting of the prepared solution (4 μL) on SPE and subsequently drying at ambient temperature. The prepared MoS_2_ NSs-SPE was used in electrochemical experiments.

The surface areas of the MoS_2_ NSs-SPE and the bare SPE were obtained by CV using 1 mM K_3_Fe(CN)_6_ at different scan rates. Using the Randles–Sevcik equation [52] for MoS_2_ NSs-SPE, the electrode surface was found to be 0.16 cm^2^ which was about 5.1 times greater than bare SPE.

### 2.4. Preparation of Real Specimens

Five AC tablets (containing 325 mg/tablet, Tehran Chemie Pharmaceutical Co., Tehran, Iran) were first powdered and then 325 mg of the powder was dissolved in water (25 mL) under ultrasonication. Next, various volumes of as-diluted solution were diluted to the mark of a 25 mL volumetric flask with PBS (pH = 7.0). The standard addition method was followed to determine the AC content.

Similarly, five INZ tablets (containing 300 mg/tablet, Tehran Chemie Pharmaceutical Co., Tehran, Iran) were first powdered and then 300 mg of the powder was dissolved in water (25 mL) under ultrasonication. Next, various volumes of as-diluted solution were diluted to the mark of a 25 mL volumetric flask with PBS (pH = 7.0). The standard addition method was followed to determine the AC content.

The instantly refrigerated urine specimens, at a certain volume (10 mL), were centrifuged at 2000 rpm for 15 min. Then, the supernatant was filtered by a 0.45 µm filter, and various volumes of it were diluted to the mark of a 25 mL volumetric flask with PBS (pH = 7.0). Next, the diluted specimens were spiked by various concentrations of INZ and AC.

## 3. Results and Discussion

### 3.1. Determination of MoS_2_ NSs Characteristics

**Figure 1:**
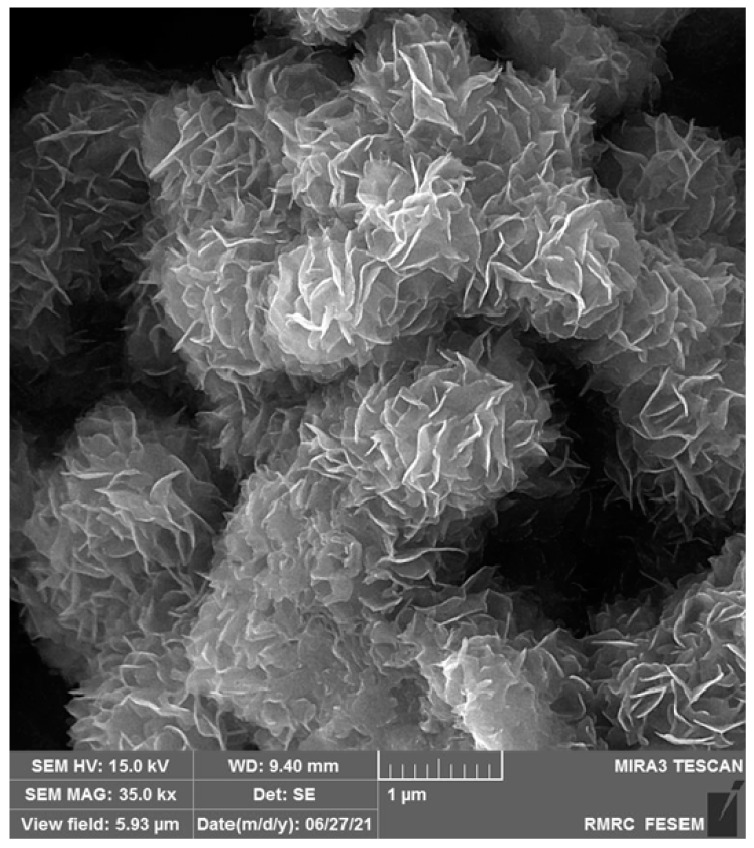
The SEM images captured for MoS_2_ NSs.

A scanning electron microscope was employed to capture images for the exploration of the morphology of MoS_2_ nanostructures (Figure 1). The MoS_2_ nanostructure is composed of thin sheets and the sheets are slightly curved and look like clusters composed of randomly assembled NSs. Moreover, SEM images show that the as-prepared MoS_2_ has a sheet-like morphology of about 12.8 nm thickness.

**Figure 2:**
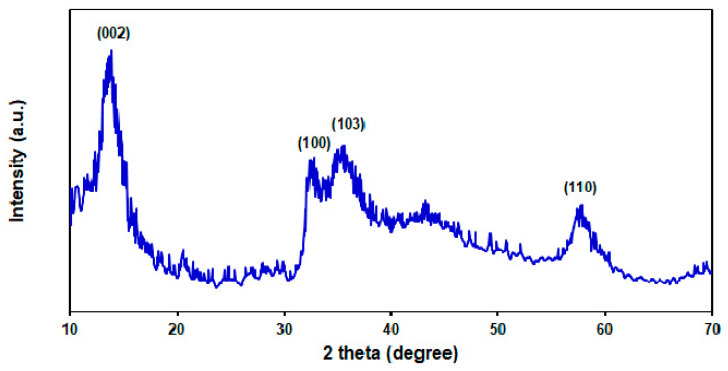
The XRD pattern captured for MoS_2_ NSs.

The XRD spectra was captured to determine the crystallographic structures of MoS_2_ NSs. Figure 2 shows the crystallite properties of MoS_2_ NSs based on the XRD spectra profiled at 57.8°, 35.3°, 32.22°, and 13.66° attributed to (110), (103), (100), and (002) crystal planes of the MoS_2_ structure, respectively, in line with the relevant standard card (JCPDS card No. 37-1492). There were no peaks related to any impurity or other phases [53].

**Figure 3:**
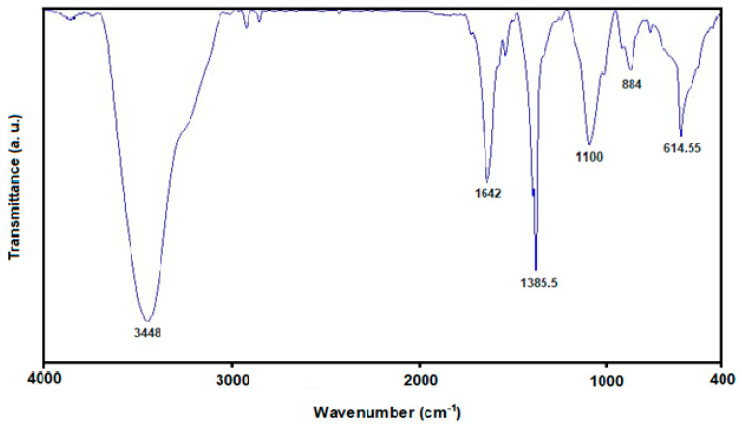
The FTIR spectrum captured for MoS_2_ NSs.

Figure 3 shows the FTIR analysis of the prepared MoS_2_ NSs, the results of which exhibited the following absorption peaks for MoS_2_ at 614.55 cm^−1^, 884 cm^−1^, 1100 cm^−1^, 1385.5 cm^−1^, 1642 cm^−1^, and 3448 cm^−1^. The peaks at 614.55 cm^−1^, 884 cm^−1^, and 1642 cm^−1^ corresponded to Mo–S and S–S bonds and Mo–O vibrations, respectively. Moreover, the peaks at 1100  cm^−1^ and 3448 cm^−1^ were related to hydroxyl stretching vibration resulting from the absorbed water molecules [54,55].

### 3.2. Electrochemical Evaluation of MoS_2_ NSs-SPE towards INZ Detection

The electrochemical determinations of INZ are significantly influenced by the solution pH. Hence, we conducted the tests to determine the pH effect on electrocatalytic behavior of MoS_2_ NSs-SPE towards INZ. The DPV was employed to study the effect of electrolyte solution pH (0.1 M PBS) under different values (2.0–9.0) in the presence of 40.0 μM of INZ at 50 mV/s on the MoS_2_ NSs-SPE. The oxidation peak current of INZ was maximum at pH 7.0, thereby selecting this value as the optimum pH in the INZ detection.

**Figure 4:**
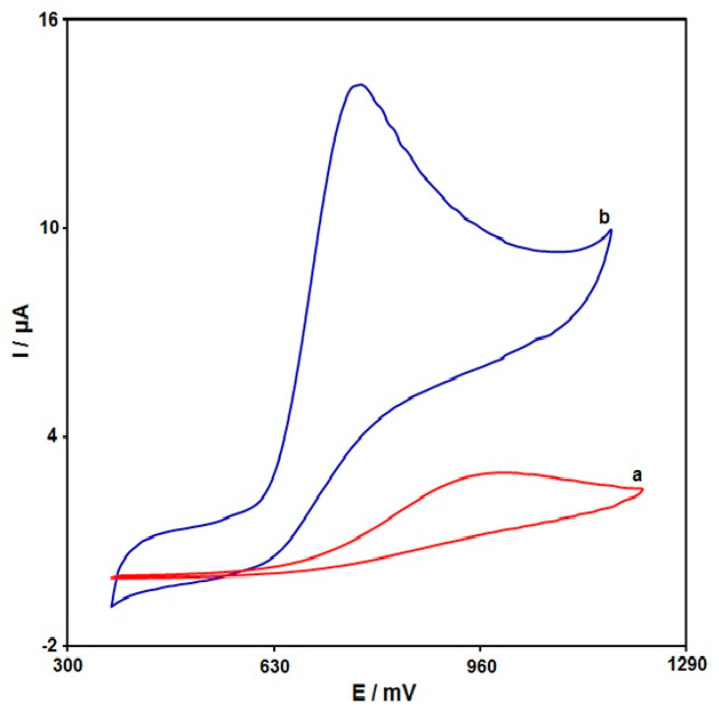
The CV curves for (curve a) unmodified SPE, and (curve b) MoS_2_ NSs-SPE in PBS (0.1 M) spiked with INZ (200.0 μM) at the scan rate of 50 mV/s.

Cyclic voltammetry was utilized to carry out all electrochemical determinations for comparison of unmodified SPE and MoS_2_ NSs-SPE in exposure to INZ (200.0 μM) at 50 mV/s (Figure 4). Findings revealed an oxidation peak, but no reduction peak, on the electrode surfaces, which means the irreversible electrochemical action of INZ on the electrodes. There was a weak and broad anodic peak current (Ipa) of INZ oxidation on the unmodified SPE at 1000 mV with 3.0 μA, underlining weak INZ oxidation on the unmodified SPE. Compared with the unmodified SPE, the Ipa of INZ obtained on the MoS_2_ NSs-SPE increases to 14.0 μA, which is almost a 4.6-fold elevation when compared with the unmodified SPE. In addition, the oxidation of INZ was seen at a lower potential when compared with the unmodified SPE. The most sensitive voltammetric response of INZ at MoS_2_ NSs-SPE was obtained from the good electrocatalytic activity of MoS_2_ NSs.

### 3.3. Effect of Scan Rate

**Figure 5:**
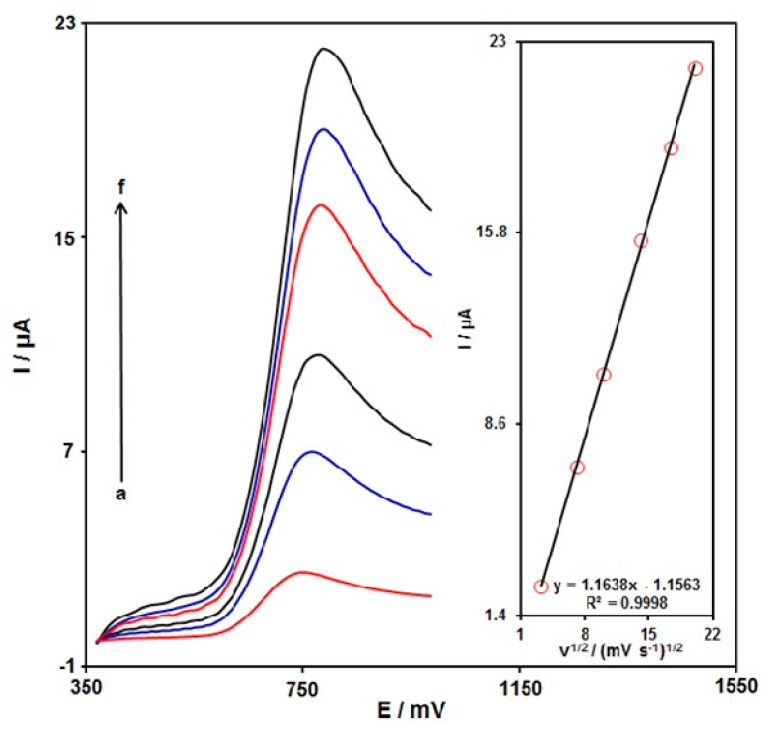
The LSV curves for INZ (100.0 μM) in PBS (0.1 M, pH 7.0) at different scan rates (10 to 400 mV/s) on MoS_2_ NSs-SPE (a–f: 10 mV/s, 50 mV/s, 100 mV/s, 200.0 mV/s, 300.0 mV/s, and 400.0 mV/s, respectively). Inset: plot of INZ oxidation peak current against square root of scan rate.

Figure 5 shows the use of LSV to determine the scan rate influence on the INZ oxidation electrocatalytically on the MoS_2_ NSs-SPE. As seen in Figure 5, the peak potential of oxidation was towards more positive directions by elevating the scan rate, which means the kinetic restriction in electrochemical process. The peak height (Ip) plot versus the scan rate square root (ν^1/2^) was linear ranging from 10 mV/s to 400 mV/s, which means the diffusion process is the main mechanism.

**Figure 6:**
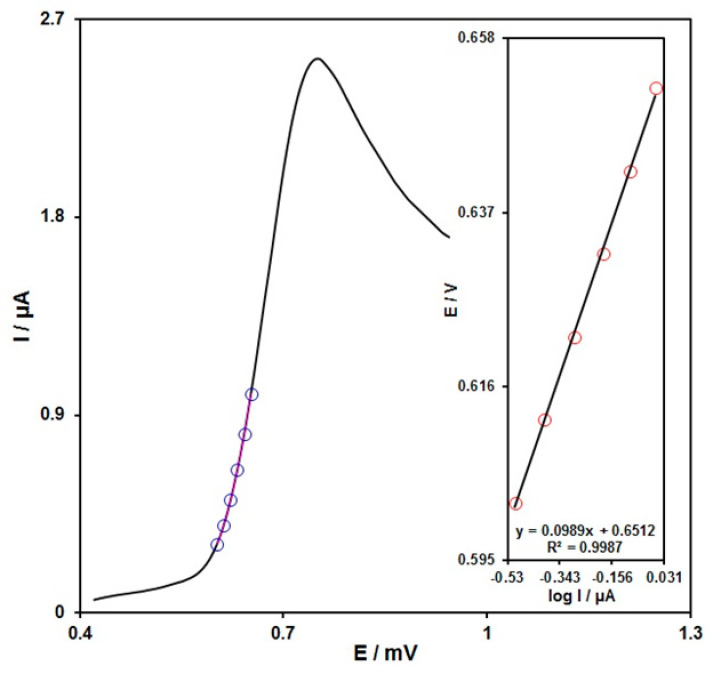
The LSV response for INZ (100.0 μM) at the scan rate of 10 mV/s. inset: Tafel plot from rising section or relevant voltammogram.

To study the rate-determining step as shown in Figure 6, the data related to the rising section of current vs. voltage curve obtained at 10 mV/s scan rate were applied to draw a Tafel plot for 100.0 μM of INZ. The linearity of E versus log I plot reveals the kinetics of the electrode process. The slope obtained from this plot was utilized to compute the electrons transfer number in the rate-determining step. Figure 6 illustrates the Tafel slope of 0.0989 V for a linear part of the plot, underlining the rate-limiting step of one-electron transfer having a transfer coefficient (α) of 0.4.

### 3.4. Chronoamperometric Measurement

**Figure 7:**
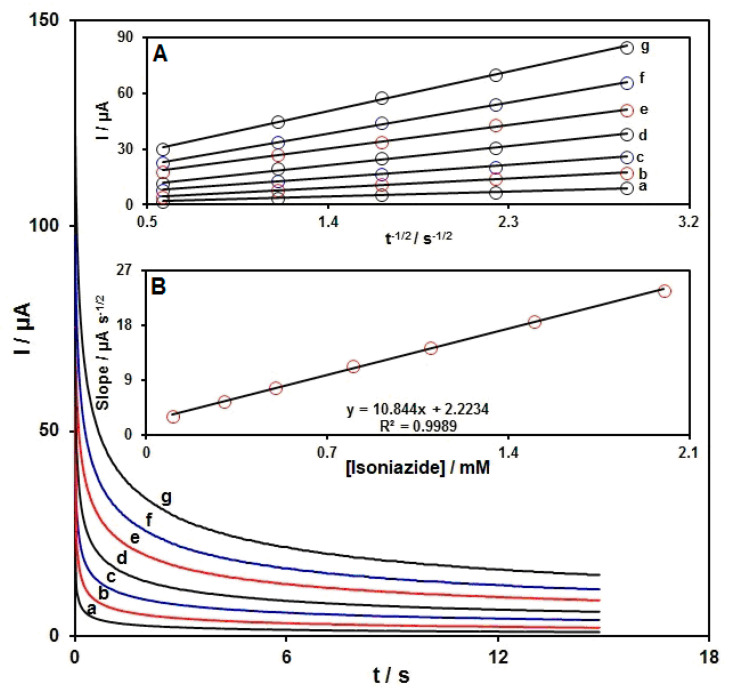
The chronoamperograms obtained on MoS_2_ NSs-SPE in PBS (0.1 M, pH = 7.0) for various INZ contents; a–g: 0.1 mM, 0.3 mM, 0.5 mM, 0.8 mM, 1.1 mM, 1.5 mM, and 2.0 mM of INZ, respectively. Inset (**A**): the plot of I against t^−1/2^ seen by chronoamperograms from a to g. Inset (**B**): the slope from the plot of straight line against INZ content.

**Figure 8:**
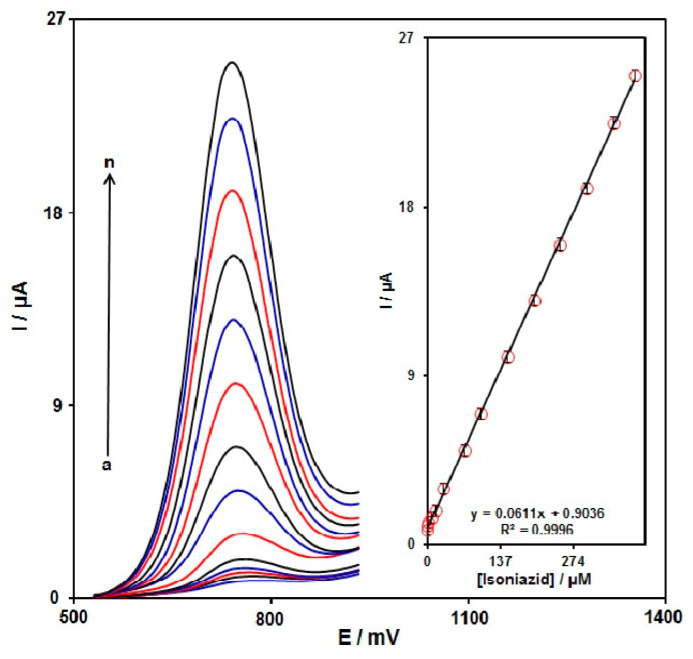
The DPV responses of INZ on MoS_2_ NSs-SPE at various INZ contents (a–n: 0.035 μM, 0.2 μM, 2.5 μM, 7.5 μM, 15.0 μM, 30.0 μM, 70.0 μM, 100.0 μM, 150.0 μM, 200.0 μM, 250.0 μM, 300.0 μM, 350 μM, and 390 μM) in PBS (0.1 M, pH = 7.0). Inset: the relationship of INZ oxidation peak currents and various INZ contents.

Chronoamperometric determinations of INZ on the MoS_2_ NSs-SPE surface were done by adjusting the potential of the working electrode at 810 mV (Figure 7). The findings from various INZ contents in PBS (at a pH value of 7.0) are depicted in Figure 8. The chronoamperometric measurement of electroactive moieties under the limited conditions of mass transfer was based on the Cottrell equation as follows:

I = nFAD ^1/2^C_b_π^−1/2^t^−1/2^

In this equation, D stands for the diffusion coefficient (cm^2^/s) and C_b_ for the bulk concentration (mol/cm^3^). The I plot against t^−1/2^ was on the basis of empirical data (Figure 7A), with the optimal fits for various INZ contents. Then, the slopes from straight lines (Figure 7A) were drawn against INZ content (Figure 7B). At last, the slope from the plot in Figure 7B and the Cottrell equation were applied to calculate the mean D value, which was 1.0 × 10^−5^ cm^2^/s.

### 3.5. DPV Detection of INZ on the Developed Sensor Surface

DPV can increase sensitivity and better features for analytical purposes. Therefore, the voltammetric sensor of MoS_2_ NSs-SPE towards INZ detection was investigated by DPV. Figure 8 shows the DPV curves of INZ with various concentrations in PBS (0.1 M, pH 7.0) solution. Based on Figure 8, the anodic peak currents exhibited linear elevation with various INZ contents (0.035–390.0 μM). The LOD was estimated 10.0 nM. The LOD and linear range of INZ at MoS_2_ NSs-SPE electrode presented in this work were compared with the reported modified electrodes and are provided in Table 1.

### 3.6. Determination of INZ in Combination with AC on MoS_2_ NSs-SPE

**Figure 9:**
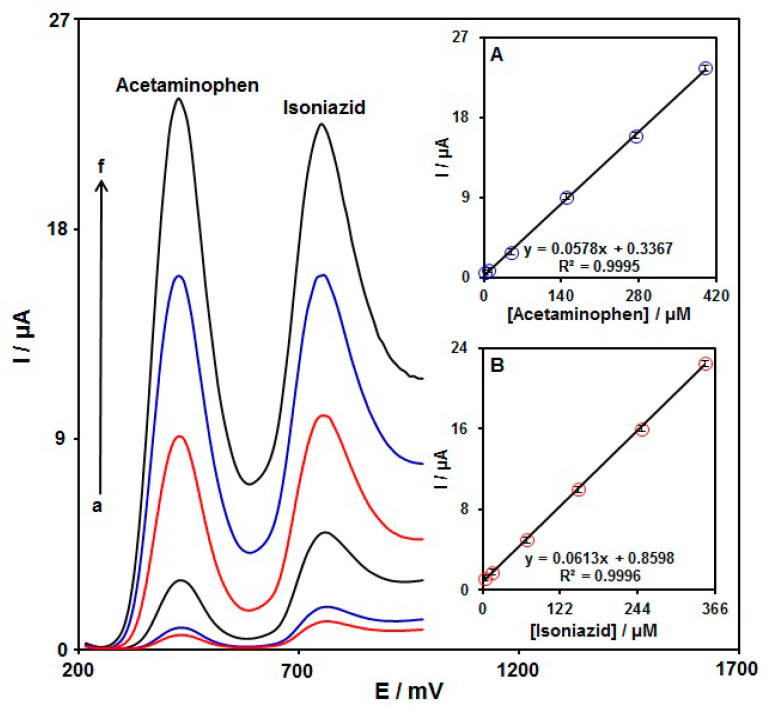
The DPVs of MoS_2_ NSs-SPE in PBS (0.1 M, pH = 7.0) with various levels of AC and INZ; a–f: 2.5 µM + 2.5 µM, 7.5 µM + 15.0 µM, 50.0 µM + 70.0 µM, 150.0 µM + 150.0 µM, 275.0 µM + 250.0 µM, and 400.0 µM + 350.0 µM of AC and INZ, respectively. Inset (**A**): the Ipa plot vs. AC concentration. Inset (**B**): the Ip plot vs. INZ concentration.

The DPVs for the detection of INZ in combination with AC via MoS_2_ NSs-SPE are presented in Figure 9. The peaks at 440 and 760 V were related to the AC and INZ oxidation, respectively. The peak current intensity was linearly elevated for both analytes by simultaneously elevating their concentrations. Figure 9 (insets A and B) shows the corresponding calibration curves for AC and INZ. The slope from the linear regression line for the calibration curve of INZ (0.0613 μA μM^−1^) was nearly equal to that without AC (0.0611 μA/μM^−1^, Section 3.5), highlighting the applicability of MoS_2_ NSs-SPE for detection of the concentrations of INZ and AC simultaneously.

### 3.7. Stability

The DPV method was used to test the stability of MoS_2_ NSs-SPE in ambient conditions. Based on the observations, the peak current of the INZ (50.0 μM) on the modified electrode maintained 96.5% of its initial current after one week, 94.7% after two weeks, and 92.6% after four weeks, which demonstrates the exceptional long-term stability of the produced sensor.

### 3.8. Interference Studies

The possible interfering effect of some potentially coexisting species with INZ in real samples was investigated. The results showed that the presence of an 800-fold concentration of Na^+^, Mg^2+^, Cl^−^, and NO_3_^−^; 500-fold concentration of glucose, Zn^2+^, Al^3+^, CO_3_^2−^, and SO_4_^2−^; and a 150-fold concentration of dopamine, ascorbic acid, uric acid, and sodium citrate caused signal changes less than ±5% for 50.0 μM INZ. However, cysteine and tryptophan with two-folds excess showed interferences. The interference experiment showed that the MoS_2_ NSs-SPE has good selectivity for determination of INZ.

### 3.9. Real Sample Analysis

The applicability of the as-developed MoS_2_ NSs-SPE towards the detection of INZ and AC was tested for INZ tablets, AC tablets, and urine specimens using the standard addition method (Table 2). According to data, the proposed electrode could preserve its efficiency for sensing INZ and AC in real specimens. As seen, reasonable recovery of INZ and AC and also satisfactory reproducibility were confirmed based on the mean relative standard deviation (RSD).

## 4. Conclusions

A novel electrochemical sensor on the basis of MoS_2_-NSs-modified SPE was established for the determination of INZ in the presence of AC. According to the findings, the MoS_2_ NSs exhibited a huge surface area and an admirable conductivity, thereby providing good electron transfer and unparalleled electrocatalytic performance in INZ and AC oxidation. There were distinct INZ and AC oxidation peaks that predisposed the detection of these two analytes concurrently on MoS_2_ NSs-SPE. A low cost of production, impressive sensitivity, and narrow limit of detection make this sensor an appropriate candidate for selective determinations of target analytes in clinical and pharmaceutical preparations. The applicability of the as-developed sensor was confirmed by determining the INZ and AC in real tablets and urine specimens, with acceptable recoveries.

## Figures and Tables

**Table 1:** Comparison of the efficiency of the MoS_2_ NSs-SPE with literature modified electrodes for INZ determination.

Electrochemical Sensor	Electrochemical Method	Linear Range	LOD	Ref.
Ordered mesoporous carbon/glassy carbon electrode	Amperometry	0.1–370 μM	83.5 nM	[8]
Electrochemically reduced graphene oxide/glassy carbon electrode	Linear sweep voltammetry	2–70 μM	0.17 ìM	[48]
Palladium nanoparticles/carbon ionic liquid electrode	Cyclic voltammetry	5–100 μM	0.47 ìM	[56]
WS_2_/carbon nanotubes/glassy carbon electrode	DPV	10–80 μM	0.24 ìM	[57]
Au–Pt core-shell nanoparticles/glassy carbon electrode	Amperometry	0.05–100 μM	29 nM	[58]
MoS_2_ NSs-SPE	DPV	0.035–390.0 μM	10.0 nM	This work

**Table 2:** Determining AC and INZ in real specimens on MoS_2_ NSs-SPE. All concentrations are in μM (*n* = 5).

Sample	Spiked		Found		Recovery (%)		R.S.D. (%)	
	AC	INZ	AC	INZ	AC	INZ	AC	INZ
AC Tablet	0	0	3.7	-	-	-	3.5	-
	2.0	5.0	5.6	5.1	98.2	102.0	2.7	3.2
	3.0	6.0	6.8	5.8	101.5	96.7	3.1	1.7
	4.0	7.0	7.9	6.9	102.6	98.6	1.9	2.9
	5.0	8.0	8.6	8.3	98.8	103.7	2.4	2.5
INZ Tablet	0	0	-	3.2	-	-	-	2.9
	5.0	2.0	4.9	5.3	98.0	101.9	2.7	3.2
	7.5	3.0	7.7	6.0	102.7	96.8	3.1	2.2
	10.0	4.0	9.9	7.5	99.0	104.2	2.1	2.4
	12.5	5.0	12.6	8.0	100.8	97.6	1.8	3.0
Urine	0	0	-	-	-	-	-	-
	5.0	4.0	5.1	3.9	102.0	97.5	1.6	3.3
	7.0	6.0	6.9	6.1	98.6	101.7	3.5	1.9
	9.0	8.0	9.3	7.9	103.3	98.7	2.7	2.4
	11.0	10.0	10.7	10.3	97.3	103.0	2.2	2.8

## Data Availability

The data presented in this study are available on request from the corresponding authors.
